# Supplementary material for: Dynamics of the formation of flat clathrin lattices in response to growth factor stimulus
Source: bioRxiv. 2025 May 23:2025.05.22.655576. Preprint. [Version 1] doi: 10.1101/2025.05.22.655576 (PMC12139753; doi:10.1101/2025.05.22.655576)
Supplement: 1 [file NIHPP2025.05.22.655576V1-supplement-1.pdf]

bioRxiv preprint doi: https://doi.org/10.1101/2025.05.22.655576; this version posted May 23, 2025. The copyright holder for this preprint (which was not certified by peer review) is the author/funder, who has granted bioRxiv a license to display the preprint in perpetuity. It is made available under aCC-BY 4.0 International license.

Supplement

1 The Turing model cannot simultaneously achieve the increase of cluster size and the increase of cluster number

Motivated by the Turing model in [56], we used the following equations to describe the dynamics of AP-2 and clathrin on the cell membrane (Figure S4A):

$$\begin{aligned}\frac{\partial[AP\text{-}2]}{\partial t} =& -\beta\Big([AP\text{-}2] - \frac{N\text{-}[AP\text{-}2]-[Clat]}{N-[AP\text{-}2]_{ss}-[Clat]_{ss}}[AP\text{-}2]_{ss}\Big) \\ &+\mu\frac{N-[AP\text{-}2]-[Clat]}{N-[AP\text{-}2]_{ss}-[Clat]_{ss}}\frac{[AP\text{-}2]}{[AP\text{-}2]_{ss}}\Big([AP\text{-}2]-[AP\text{-}2]_{ss}\Big)+D_{AP\text{-}2}\triangle[AP\text{-}2] \qquad \text{(S1)}\\ \frac{\partial[Clat]}{\partial t} =& -b\Big([Clat]-\frac{[AP\text{-}2]}{[AP\text{-}2]_{ss}}\frac{N-[AP\text{-}2]-[Clat]}{N-[AP\text{-}2]_{ss}-[Clat]_{ss}}[Clat]_{ss}\Big)\\ &-m_1\frac{N-[AP\text{-}2]-[Clat]}{N-[AP\text{-}2]_{ss}-[Clat]_{ss}}\Big([Clat]-[Clat]_{ss}\Big)\\ &+m_2\frac{N-[AP\text{-}2]-[Clat]}{N-[AP\text{-}2]_{ss}-[Clat]_{ss}}\frac{[Clat]}{[Clat]_{ss}}\Big([AP\text{-}2]-[AP\text{-}2]_{ss}\Big)+\widetilde{D_{Clat}}\triangle[Clat] \qquad \text{(S2)}\end{aligned}$$

where $[AP\text{-}2]$ and $[Clat]$ denote the concentrations of AP-2 and clathrin on the cell membrane, respectively. All terms in Equations (S1) and (S2) are the same as those in [56] except the diffusion terms. The diffusion terms $D_{AP-2}\triangle[AP\text{-}2]$ and $\widetilde{D_{Clat}}\triangle[Clat]$ in Equations (S1) and (S2) are simpler than that in [56], but the Turing pattern still can be maintained. Four mechanisms that are key to Turing pattern formation are as follows: 1) the enhanced recruitment of AP-2 if one AP-2 has already bound to the cell membrane (the curved arrow in Figure S4A); 2) the recruitment of clathrin to the cell membrane caused by AP-2 (the arrow from AP-2 to clathrin in Figure S4A); 3) the steric repulsion between clathrin and AP-2 (the arrow from clathrin to AP-2 in Figure S4A); 4) much slower diffusion coefficient of AP-2 compared to that of clathrin. In the first two mechanisms, the reaction strength is denoted by μ and m_2. For the third mechanism, it is achieved by multiplying $\frac{N-[AP\text{-}2]-[Clat]}{N-[AP\text{-}2]_{ss}-[Clat]_{ss}}$ to each reaction term: the larger the $[AP\text{-}2]+[Clat]$ is, the smaller the $\frac{N-[AP\text{-}2]-[Clat]}{N-[AP\text{-}2]_{ss}-[Clat]_{ss}}$ is, leading to less recruitment events. Here, N is set to be a large enough number to ensure $[AP\text{-}2]+[Clat]\leq N$ hold all the time, and the subscript ss denotes the homogeneous steady-state value. As for the fourth mechanism, we set the ratio of diffusion coefficients between AP-2 and clathrin $D_{AP\text{-}2}/\widetilde{D_{Clat}}$ as 0.0377 in our simulations.

The values of kinetic parameters in Equations (S1) and (S2) are listed in Table S3, which are determined by searching previous clathrin models or the requirement of Turing instability. According to the clathrin model in [30], the dissociation rate of AP-2 and PIP_2 is 1 s^{-1}, and the dissociation rate of clathrin and AP-2 is 0.03 s^{-1}. Therefore, we set the dissociation rate of AP-2 and cell membrane β to be 1 s^{-1}, and the dissociation rate of clathrin and cell membrane b 0.03 s^{-1}. Besides, the values of N, $[AP\text{-}2]_{ss}$ and $[Clat]_{ss}$ were chosen to match the scale of AP-2 in [30] ($\sim$ 361 copies/μm^2). Other kinetic parameters were determined by the constraints of Turing instability, such as μ, m_1, m_2, D_{AP-2} and $\widetilde{D_{Clat}}$. It should be noted that the value of D_{AP-2} is 1% of AP-2 translational diffusion constant on the membrane in [30], and that the value of $\widetilde{D_{Clat}}$ is also 1% of clathrin translational diffusion constant in [30]. This rescaling of the diffusion constant ensures the correct size of each clathrin cluster.

We first tested whether the Turing model can capture the experimental observations of FCL

bioRxiv preprint doi: https://doi.org/10.1101/2025.05.22.655576; this version posted May 23, 2025. The copyright holder for this preprint (which was not certified by peer review) is the author/funder, who has granted bioRxiv a license to display the preprint in perpetuity. It is made available under aCC-BY 4.0 International license.

dynamics after the stimulus of EGF. By simulating the Turing model, we obtained a stable pattern, corresponding to the system without stimulus (the first plot in Figure S4B). Then, we increased the association rate of AP-2 and membrane μ by 80% to mimic the effect of adding EGF. Under this change, the system experiences two stages: (1) in [0, 15 seconds], each clathrin cluster grows in size but no new clusters emerge (Figure S4B); (2) after 15 seconds, each large clathrin cluster disassembles into small clusters, leading to the increase in the number of clusters and decrease in the size of each cluster. To make the trend of cluster size and cluster number more clear, we used the image processing tool to obtain the boundary of each cluster and then calculated the cluster size and cluster number quantitatively (Figure S4C). It can be seen that the increase in cluster size and the increase in total cluster number occur at different time intervals, i.e., [0 15 seconds] and after 15 seconds, respectively. However, as we can see from the experimental data (Figure 1E), the increase in the size and that in the number happens simultaneously. Taken together, the Turing model cannot capture the FLC dynamics under the EGF stimulus.

bioRxiv preprint doi: https://doi.org/10.1101/2025.05.22.655576; this version posted May 23, 2025. The copyright holder for this preprint (which was not certified by peer review) is the author/funder, who has granted bioRxiv a license to display the preprint in perpetuity. It is made available under aCC-BY 4.0 International license.

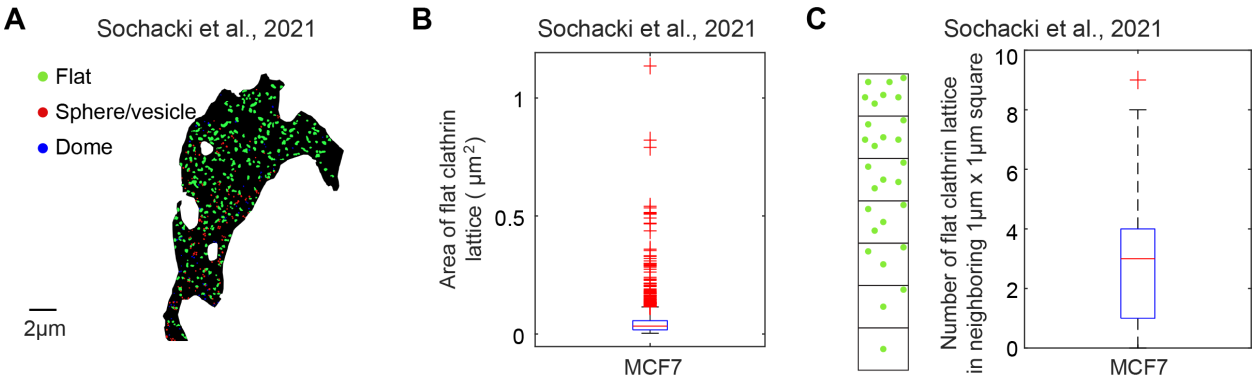

Figure S1: The FCL distributions in MCF7 cells. (A-C) The same plots as those in Figure 1B-D except the cell type.

bioRxiv preprint doi: https://doi.org/10.1101/2025.05.22.655576; this version posted May 23, 2025. The copyright holder for this preprint (which was not certified by peer review) is the author/funder, who has granted bioRxiv a license to display the preprint in perpetuity. It is made available under aCC-BY 4.0 International license.

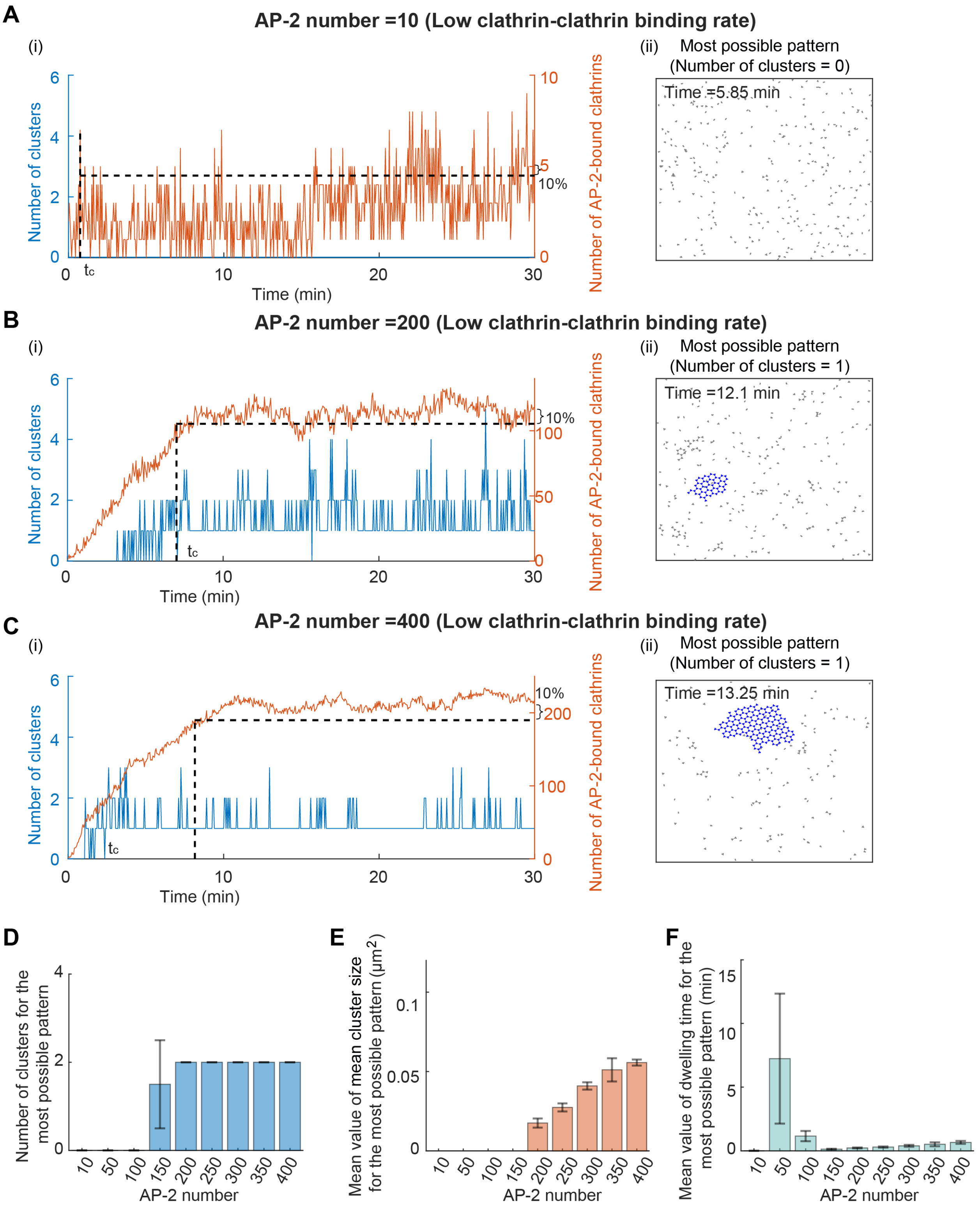

Figure S2: The FCL exhibits the phase transition from no cluster to one giant cluster when the AP-2 number increases while maintaining a low clathrin-clathrin binding rate. (A-F) The same plots as those in Figure 3 except that a low clathrin-clathrin binding rate is used.

bioRxiv preprint doi: https://doi.org/10.1101/2025.05.22.655576; this version posted May 23, 2025. The copyright holder for this preprint (which was not certified by peer review) is the author/funder, who has granted bioRxiv a license to display the preprint in perpetuity. It is made available under aCC-BY 4.0 International license.

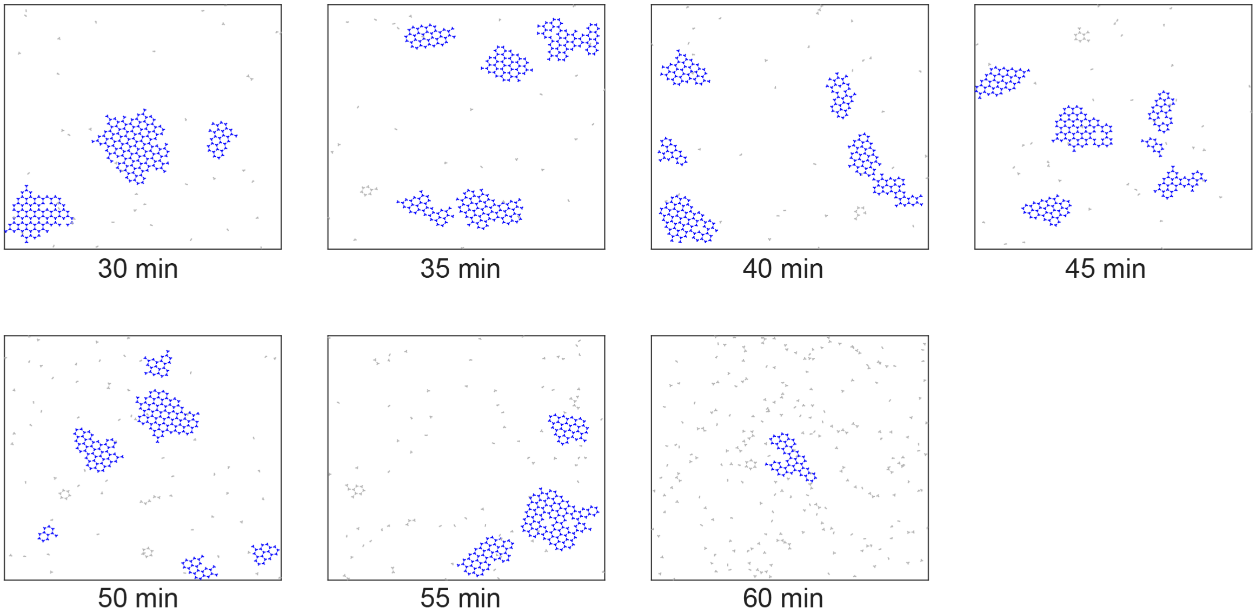

Figure S3: Simulated FCL dynamics after 30 minutes of EGF stimulus when kinetic parameters change follow the way in Figure 6B(4).

bioRxiv preprint doi: https://doi.org/10.1101/2025.05.22.655576; this version posted May 23, 2025. The copyright holder for this preprint (which was not certified by peer review) is the author/funder, who has granted bioRxiv a license to display the preprint in perpetuity. It is made available under aCC-BY 4.0 International license.

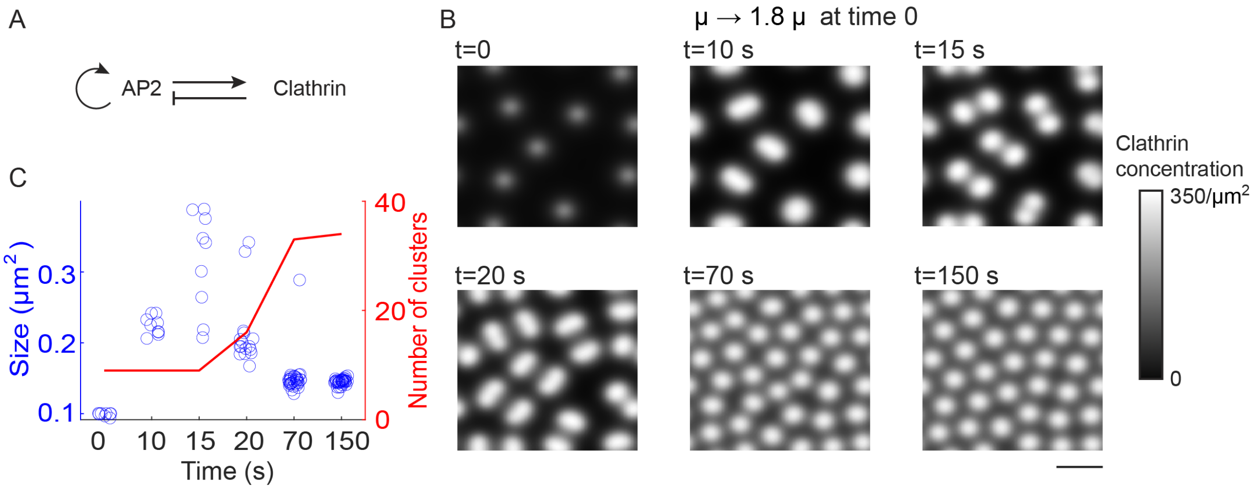

Figure S4: The Turing model cannot simultaneously achieve the increase of cluster size and the increase of cluster number. (A). Schematic of reactions between the AP-2 and the clathrin. The AP-2 improves the recruitment of itself and the clathrin to the cell membrane. Once bound to the cell membrane, the clathrin inhibits the accumulation of the AP-2 on the cell membrane due to steric repulsion. (B). The snapshots of clathrin clusters after increasing the association rate of AP-2 and membrane μ by 80% at time 0. The first plot corresponds to the Turing pattern with parameters in Table S3. Scale bar is 1 $\mu\mathrm{m}$. (C). The size for each clathrin cluster (left axis) and the total number of clathrin clusters (right axis) at the time points in (B). The increase in the clathrin cluster size only occurs between 0 and 15 seconds, while the increase of the total number of clathrin clusters occurs after 15 seconds.

bioRxiv preprint doi: https://doi.org/10.1101/2025.05.22.655576; this version posted May 23, 2025. The copyright holder for this preprint (which was not certified by peer review) is the author/funder, who has granted bioRxiv a license to display the preprint in perpetuity. It is made available under aCC-BY 4.0 International license.

Table S1: Parameters used in the particle-based model (from [30])

**Parameters**	**Explanations**
AP-2-clathrin binding rate $k_{AP\text{-}2\cdot Clat}$	0.0012 $\mu\mathrm{M}^{-1}\mathrm{s}^{-1}$
Clathrin-clathrin binding rate without AP-2 $k_{Clat\cdot Clat}$	0
AP-2 diffusion coefficient $D_{AP\text{-}2}$	0.5 $\mu\mathrm{m}^2/\mathrm{s}$
**Parameters used in Figure 2**	
AP-2-clathrin dissociation rate $d_{AP\text{-}2\cdot Clat}$	0.003 s^{-1}
Clathrin-clathrin binding rate with AP-2 $k_{(AP\text{-}2\cdot)Clat\cdot Clat}$	$20 \times 1.83\mu\mathrm{M}^{-1}\mathrm{s}^{-1}$
Clathrin diffusion coefficient D_{Clat}	13 $\mu\mathrm{m}^2/\mathrm{s}$
AP-2 number	150
Clathrin-clathrin dissociation rate $d_{Clat\cdot Clat}$	10 s^{-1}
**Parameters used in Figure 3**	
$d_{AP\text{-}2\cdot Clat}$	0.003 s^{-1}
$k_{(AP\text{-}2\cdot)Clat\cdot Clat}$	$50 \times 1.83\mu\mathrm{M}^{-1}\mathrm{s}^{-1}$
D_{Clat}	13 $\mu\mathrm{m}^2/\mathrm{s}$
AP-2 number	[10, 50, 100, 150, 200, 250, 300, 350, 400]
$d_{Clat\cdot Clat}$	10 s^{-1}
**Parameters used in Figure 4**	
$d_{AP\text{-}2\cdot Clat}$	0.003 s^{-1}
$k_{(AP\text{-}2\cdot)Clat\cdot Clat}$	$[1, 25, 50, 75, 100] \times 1.83\mu\mathrm{M}^{-1}\mathrm{s}^{-1}$
D_{Clat}	13 $\mu\mathrm{m}^2/\mathrm{s}$
AP-2 number	100
$d_{Clat\cdot Clat}$	10 s^{-1}
**Parameters used in Figure 5**	
$d_{AP\text{-}2\cdot Clat}$	0.003 s^{-1}
$k_{(AP\text{-}2\cdot)Clat\cdot Clat}$	$50 \times 1.83\mu\mathrm{M}^{-1}\mathrm{s}^{-1}$
D_{Clat}	$[1, 1/5, 1/10, 1/20, 1/30, 1/40] \times 13\mu\mathrm{m}^2/\mathrm{s}$
AP-2 number	100
$d_{Clat\cdot Clat}$	10 s^{-1}
**Parameters used in Figure 6A**	
$d_{AP\text{-}2\cdot Clat}$	0.0003 s^{-1}
$k_{(AP\text{-}2\cdot)Clat\cdot Clat}$	$1.83\mu\mathrm{M}^{-1}\mathrm{s}^{-1}$
D_{Clat}	$13\mu\mathrm{m}^2/\mathrm{s}$
AP-2 number	100
$d_{Clat\cdot Clat}$	10 s^{-1}

bioRxiv preprint doi: https://doi.org/10.1101/2025.05.22.655576; this version posted May 23, 2025. The copyright holder for this preprint (which was not certified by peer review) is the author/funder, who has granted bioRxiv a license to display the preprint in perpetuity. It is made available under aCC-BY 4.0 International license.

Table S2: Relative coordinate for clathrin

First binding site to clathrin	$(3, 3\sqrt{3}, 0)$
Second binding site to clathrin	$(3, -3\sqrt{3}, 0)$
Third binding site to clathrin	$(-6, 0, 0)$
Binding site to AP-2	$(0, 0, -3)$

Table S3: Parameters used in Turing model

**Parameters**	**Explanations**	**Values**	**Ref.**
**Parameters used in the Turing model**			
β	AP-2 & membrane dissociation rate	1 s^{-1}	[30]
μ	AP-2 & membrane association rate	1.4 s^{-1}	Fitted
b	Clathrin & membrane dissociation rate	0.03 s^{-1}	[30]
m_1	Clathrin & membrane dissociation rate caused by unspecified molecules	0.8s^{-1}	Fitted
m_2	Recruitment rate of clathrin by AP-2	10 s^{-1}	Fitted
D_{AP-2}	Diffusion coefficient of AP-2	0.049 $\mu\mathrm{m}^2\mathrm{s}^{-1}$	1/100 of the value in [30]
D_{Clat}	Diffusion coefficient of clathrin	1.3 $\mu\mathrm{m}^2\mathrm{s}^{-1}$	1/100 of the value in [30]
N	Maximal total concentration	500 $\mu\mathrm{m}^{-2}$	Same scale as in [30]
$[AP\text{-}2]_{ss}$	Homogeneous steady-state value of [AP-2]	25 $\mu\mathrm{m}^{-2}$	Same scale as in [30]
$[Clat]_{ss}$	Homogeneous steady-state value of [Clat]	25 $\mu\mathrm{m}^{-2}$	Same scale as in [30]
